# A Roadmap towards Precision Periodontics

**DOI:** 10.3390/medicina57030233

**Published:** 2021-03-03

**Authors:** Mia Rakic, Natasa Pejcic, Neda Perunovic, Danilo Vojvodic

**Affiliations:** 1 ETEP (Etiology and Therapy of Periodontal Diseases) Research Group, Faculty of Dentistry, University Complutense of Madrid, Pza. Ramón y Cajal s/n, 28040 Madrid, Spain; 2 Department of Preventive and Pediatric Dentistry, Faculty of Dental Medicine, University of Belgrade, 11000 Belgrade, Serbia; natasadpejcic@yahoo.com; 3 Department of Periodontology and Oral Medicine, Faculty of Dental Medicine, Dr Subotica 8, University of Belgrade, 11000 Belgrade, Serbia; neda.perunovic@stomf.bg.ac.rs; 4 Institute for Medical Research, Military Medical Academy, University of Defense, 11000 Belgrade, Serbia; vojvodic.danilo@gmail.com

**Keywords:** periodontitis, biomarkers, precision medicine, diagnosis, algorithms, machine learning

## Abstract

Periodontitis is among the most common health conditions and represents a major public health issue related to increasing prevalence and seriously negative socioeconomic impacts. Periodontitis-associated low-grade systemic inflammation and its pathological interplay with systemic conditions additionally raises awareness on the necessity for highly performant strategies for the prevention and management of periodontitis. Periodontal diagnosis is the backbone of a successful periodontal strategy, since prevention and treatment plans depend on the accuracy and precision of the respective diagnostics. Periodontal diagnostics is still founded on clinical and radiological parameters that provide limited therapeutic guidance due to the multifactorial complexity of periodontal pathology, which is why biomarkers have been introduced for the first time in the new classification of periodontal and peri-implant conditions as a first step towards precision periodontics. Since the driving forces of precision medicine are represented by biomarkers and machine learning algorithms, with the lack of periodontal markers validated for diagnostic use, the implementation of a precision medicine approach in periodontology remains in the very initial stage. This narrative review elaborates the unmet diagnostic needs in periodontal diagnostics, the concept of precision periodontics, periodontal biomarkers, and a roadmap toward the implementation of a precision medicine approach in periodontal practice.

## 1. Introduction

With constant population growth and increased human lifespan, oral health strategies that have massively decreased the rate of tooth loss have expectedly resulted in an outbreak of periodontal diseases. Periodontitis represents a major public health problem [1] since this highly prevalent chronic disease negatively affects oral and systemic health; it has a negative impact on oral-health-related quality of life (OHRQoL) by causing impaired function and esthetics, while representing a second main cause of tooth loss in adults, collectively increasing health care costs [2,3,4,5]. For these reasons, immense efforts are continually invested in the improvement of periodontal strategies for prevention and treatment, having as their objective a reduction of the global periodontal burden with all its negative socio-economic impacts. Diagnosis represents the backbone of successful periodontal treatment since the entire treatment plan, prognosis, and maintenance directly depend on the quality and precision of periodontal diagnosis. The crucial importance of accurate periodontal diagnostics extends far beyond clinical practice, since the quality and performance of research studies aiming to improve periodontal management strategies indeed hinge on the accuracy of the diagnostic indicators used. Since periodontal diagnostics is still based on clinical and radiological parameters providing limited therapeutic guidance, the use of biomarkers has been introduced for the first time within the new classification of periodontal and peri-implant conditions as a first step towards the adoption of precision medicine concepts in periodontology [6]. Alongside this, the use of biomarkers in periodontal research is officially the subject of recommendation within guidelines for clinical research in the domain of oral and maxillofacial regeneration [7]. Unfortunately, there is still no biomarker validated for diagnostic use in periodontology, and since biomarkers are the driving force of precision medicine, the implementation of a precision medicine approach remains substantially delayed.

This review elaborates the unmet diagnostic needs in periodontal diagnostics, the concept of precision periodontics, periodontal biomarkers, and a pipeline for accelerated implementation of the precision medicine approach in periodontal practice.

## 2. Challenging Aspects of Periodontal Diagnosis and Unmet Diagnostic Needs

Periodontitis is a chronic multifactorial inflammatory disease triggered by dysbiotic biofilms and characterized by periodontal tissue destruction, clinically manifesting as clinical attachment loss (CAL), the presence of periodontal pocketing, gingival bleeding, and radiological signs of alveolar bone loss [3]. Technological progress in biomedicine, particularly regarding high-throughput methods, highly sensitive diagnostic platforms, and machine learning algorithms, together with tremendous progress in periodontal research, has completely changed the face of periodontal pathogenesis and clearly revealed the limitations of the standard clinical approach in providing highly reliable and patient-specific diagnostic information. The human body relies on the principle of dynamic homeostasis regulated by compiled mechanisms of positive and negative feedback [8], and the new breakthrough technologies enable the objective portrayal of this principle, raising awareness about the necessity of a comprehensive assessment of a diverse parameter panel for reliable and patient-specific diagnosis. The previous periopathogen-centered theories have been replaced by the key-stone pathogen hypothesis of periodontal disease, emphasizing the critical role of dramatic compositional changes within the periodontal microbiome triggered by key-stone periopathogens, rather than individual periopathogens [9,10]. The suppression of aggressive periodontitis from the classification of periodontal disease is the best example, since highly sensitive molecular methods demonstrated that Aggreggatibacter actinomycetemcomitans, initially considered a form-specific pathogen, was present in less than 50% of aggressive periodontitis cases and showed a similar distribution between healthy and diseased patients. In the context of periodontal treatment, advances in biofilm research have revealed the complexity of biofilm structures, while the most prominent discoveries with clinical relevance are the importance of targeting the primary and secondary colonizers within preventive strategies. Additionally, the interference between biofilm-embedded bacteria with standard antimicrobial treatments and routine antibiograms has been demonstrated [11], emphasizing the need for anti-biofilm approaches and the use of advanced methods for microbial sensitivity, such as culturomics.

Moreover, studies have demonstrated the inflammophilic character of pathogens [12], providing new insights regarding pathological conversion between periodontal conditions and the role of non-periodontal pro-inflammatory factors in periodontal pathology, while announcing immunological dysbiosis as a critical determinant of periodontal diseases [13]. It is currently established that dysbiotic biofilms remain necessary but not sufficient to trigger periodontal diseases, while reinforced interactions between a dysbiotic microbiome and dysregulated inflammation are a hallmark of periodontal disease [14]. This particularly facilitated the understanding, identification, and confirmation of periodontal risk factors, such as the impact of viral and fungal species [15,16], systemic conditions, bad habits, hormonal changes, aging, and many other factors relying on inflammatory processes and their respective roles in local immunological breakdown and the deterioration of periodontal conditions. Finally, it is considered that the immunophenotype plays an important role in the severity of periodontitis [17], since it is considered that individuals with an overreactive genetic predisposition excessively react even to small amounts of bacterial biofilms, and this is particularly associated with periodontitis Stage 3 (previously called severe periodontitis) [6] that affects up to 20% of the population [18]. Periodontitis Stage 3 is a special focus of public health and periodontal research since standard treatment protocols frequently fail to arrest progressive periodontal destruction, while such patients exhibit low-grade systemic inflammation [19,20]. When considering all these facts as a whole, it is clear that the multifactorial complexity of periodontal pathology exceeds the capacity of a standard clinical diagnosis in providing accurate diagnosis and requires biomarker-supported diagnostics [21].

In the context of the aforementioned, the unmet diagnostic needs in periodontal diagnostics and related requests for a precision approach are as follows:

- Predictors and markers for periodontitis onset;
- Markers for disease activity and progression;
- Prognostic markers for the treatment of periodontitis;
- Prognostic markers for tailored treatment.

## 3. Principles of Precision Periodontics

Precision medicine is grounded on a combination of clinical parameters and biological markers reflecting the underlying biological processes; this enables highly reliable prediction of periodontal disease susceptibility, early diagnosis, prognosis, and planning of the most effective and safe treatment strategy meeting individual patient needs [22]. In vitro diagnostics (IVD) affects about 60% of all medical decisions today, with a focus on unmet diagnostic needs in the course of improving patient-specific diagnoses and providing optimal treatment plans for high-standard healthcare deliverance. IVD is a critical source of objective information concerning a specific disease profile and related aggravating/risk factors to be accounted for in optimal prevention or disease management. Finally, IVD reduces overall healthcare costs by preventing unnecessary disease occurrence and avoiding unadapted management strategies. Thus, IVD empowers clinicians to base decisions on highly specific and accurate diagnostic information and to customize a management strategy to fit individual patient needs. Another hallmark of precision medicine is that such an approach actually represents a point where a plethora of various biomedical fields—such as genetics, microbiology, immunology, biochemistry, histology, and pathology—meet clinical practice, by compiling the knowledge into a highly performant management strategy. So, given the multifactorial nature of diseases targeted by precision medicine [23], such an approach implies a comprehensive assessment of a broad panel of anamnestic, clinical, and biological parameters that are implemented in a highly accurate diagnostic information pool via machine learning algorithms [24,25]. Machine learning algorithms have the capacity to cross-analyze unlimited numbers of clinical and biological parameters while identifying a panel of critical determinants within highly specific patterns, which are further integrated into accurate and interpretable diagnostic information [25].

## 4. Periodontal Biomarkers as a Fundament for Precision Periodontics

**Figure 1:**
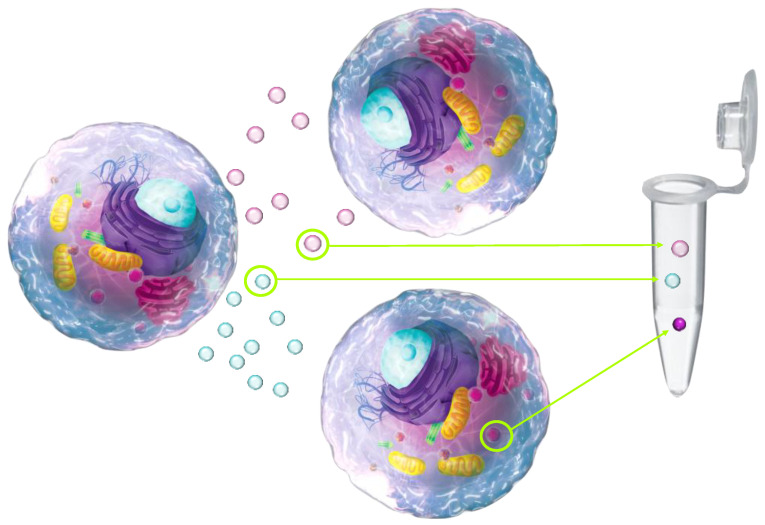
The concept of biomarkers relies on the measurement of regulators or byproducts of the biological processes of interest, starting from the cellular level, through intercellular interactions, to complex interplay within the tissues, organs, and organic systems. Such a diagnostic concept provides objectively measurable diagnostic information on the target biological processes in real time, compensating most common limitations of clinical parameters. With tremendous progress in biomedical technologies, practically any component of a biological process is measurable.

A biomarker is “a characteristic that is objectively measured and evaluated as an indicator of normal biological processes, pathogenic processes or pharmacological responses to a therapeutic intervention” [26]. Actually, biomarkers represent the extracted regulators or byproducts of a target biological process that are measured in vitro, allowing for objective measurement of the ongoing biological processes/effects in real time. Thanks to outstanding progress in biomedical research, it is currently possible to measure practically any element of a biological process regulated by complex intercellular communication via active biological substances synthetized and released from the cells and tissues as an outcome of genetical control mechanisms modulated in response to local and epigenetic factors (Figure 1).

The great advantage of periodontal marker assessment is the accessibility of diagnostic specimens that can be obtained by lowly invasive and inexpensive procedures. Hence, dental plaque as a diagnostic specimen for microbiological assessment can be easily collected by means of sub-gingival swab using standardized precut methylcellulose filter strips or using universal endodontic paper of higher caliber (>30) [27,28]. Furthermore, the gingival crevicular fluid (GCF), the precious capillary fluid derived by serum transudation into the gingival sulcus that, under pathological conditions, converts to inflammatory exudate, qualitatively corresponds to a biopsy and has outstanding diagnostic capacity [29]. Another advantage of the oral environment is that even DNA-containing specimens for genetic markers can be collected using a buccal swab without requiring venipuncture. Finally, soft and bone tissue biopsies can be easily retrieved during routine non-surgical or surgical procedures.

**Figure 2:**
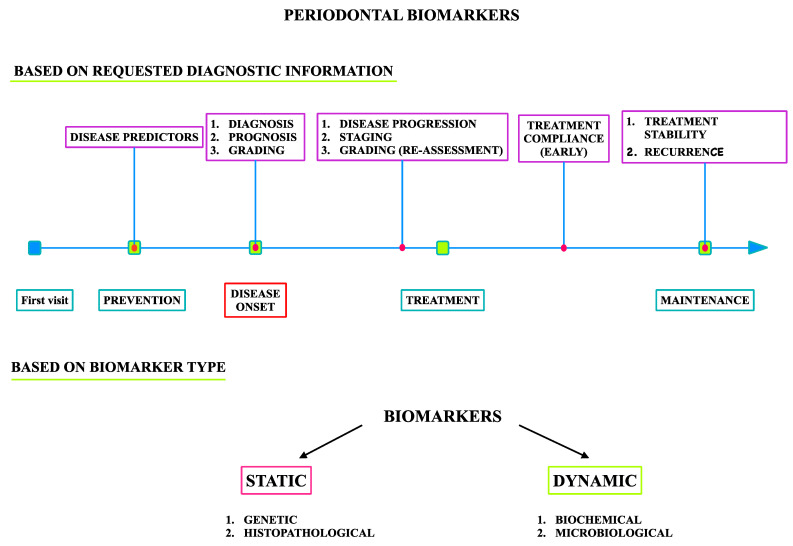
Biomarkers in periodontology.

Biomarkers in periodontology can be classified based on the requested diagnostic information and based on the biological type (Figure 2) appropriate for the clinical strategy in order to provide diagnostic information specific to each clinical phase.

Biomarkers in periodontology can be categorized as follows (Figure 2):

- Predictive markers measured in healthy individuals in the disease prevention stage;
- Diagnostic markers of disease onset;
- Prognostic markers for the assessment of disease progression, stage, and grade in the treatment planning phase;
- Diagnostic markers and surrogate endpoints used to estimate patient compliance with the administered treatment, stability of the therapy results, and disease activity in the maintenance phase.

Predictive markers are used before disease occurrence for the identification of risk factors and estimation of the overall patient risk, aiming at adjustment of the screening protocol and related modification of risk factors for optimal disease prevention. For this purpose, static markers are usually used; these do not change over time and are typically genetic markers. Single-nucleotide polymorphisms (SNPs) are certainly the most studied class of genetical markers in periodontology [30], presenting as variations in single base-pair components of DNA that determine host responsiveness to environmental challenges, such as infection. In brief, patients with specific immunophenotypes are considered to be more prone to impaired elimination of periodontal pathogens and/or to inflammatory overreaction to pathogens, resulting in excessive periodontal destruction. For this reason, the cytokines and immunoreceptors responsible for pathogen recognition remain in the spotlight of genetic studies. It has been proposed that SNPs in the IL1β, IL1RN, FcγRIIIb, VDR, and TLR4 genes may underlie susceptibility to more destructive forms of periodontitis, while polymorphisms in the IL1B, IL1RN, IL6, IL10, VDR, CD14, TLR4, and MMP1 genes might be responsible for general susceptibility to chronic periodontitis [31]. However, inconsistencies in case definition, study design, and population restriction, as well as small sample sizes, among the studies have substantially compromised the process of the validation of genetic markers in periodontology.

Prognostic markers are measured when disease occurs; they do not need to change over time, and they serve to estimate disease characteristics, stage, and grade, which are indispensable for accurate prognostics of the progression pattern and responsiveness to different treatment protocols. The most frequently used are genetic markers [32]. Therefore, prognostic markers should guide the clinician in the process of treatment planning to mitigate aggravating factors and minimize disease complications, in the selection of a suitable treatment protocol, and in setting the maintenance regimen for optimal treatment stability. Diagnostic markers comprise a wide group of indicators able to disclose disease onset, disease activity, and related disease progression, usually represented by fast-response biochemical and microbiological markers. A specific subset of diagnostic markers includes surrogate endpoints intended for estimation of the patient’s compliance to the administered treatment. These groups of biomarkers mostly comprise soluble inflammatory, soft tissue, and bone turnover markers (BTMs) [33,34]. The inflammatory biomarkers in periodontology are represented by pro- and anti-inflammatory cytokines, host-derived enzymes, and markers of oxidative stress. Since these markers are elevated in both gingivitis and periodontitis, they are preferably used to estimate disease activity, progression, and compliance with administered treatment. The most investigated markers in periodontology are IL-1β, IFNγ, and TNFα from the T-helper (Th)-1 sub-family; IL-6, IL-4, and IL-10 from the Th-2 sub-family; IL-17 from the Th-17 sub-family; and IL-8. In general, Th-1 and Th-17 markers are generally increased in active periodontitis and usually decrease following treatment, while Th-2 markers seems to be slightly less specific than Th-1 and Th-17 [35,36,37,38]. A disbalance between reactive oxygen species (ROS) and antioxidants is a pathological characteristic of periodontal destruction that is frequently exploited for diagnostic purposes in periodontology. The markers that show a specific profile and good treatment responsiveness to periodontal treatment are malondialdehyde, nitric oxide, total oxidant status, total antioxidant capacity, and 8-hydroxy-deoxyguanosine measured in saliva, while GCF profiles are slightly less specific [39,40].

Soft tissue markers are used for monitoring soft tissue degradation and regeneration, and matrix metalloproteinases (MMPs) and growth factors are the most repurposed markers used in periodontology [34]. So far, MMP-8 remains the most promising soft tissue marker in periodontology; however, further validation studies are required. BTMs are considered the most important markers in periodontology to indicate inflammatory osteoclastogenesis onset and activity within periodontitis onset and activity, respectively. The system of receptor activator nuclear kappa B (RANK) is a major focus of BTM research in periodontology since this receptor is located on pre-osteoclasts and mature osteoclasts, with its ligand (RANKL) and respective antagonist (osteoprotegerin, OPG) also forming the regulatory triad of pathological bone resorption [41]. Thus, the RANKL/OPG relative ratio was proposed as a promising marker of periodontitis onset, but its diagnostic value in the assessment of disease activity has not yet been established [33,42]. The pyridinoline cross-linked carboxyterminal telopeptide of type I collagen (ICTP) is a byproduct of bone type I collagen degradation that is a highly specific marker of bone resorption, significantly increased in periodontitis and strongly correlated to clinical periodontal parameters; it is modestly responsive in periodontal treatment, but with promising predictive capacity for future alveolar bone loss [43,44]. Cathepsin-K, calprotectin, and osteocalcin also show a promising capacity for periodontitis diagnosis and predicting treatment outcomes [45,46]. In the context of microbiological markers, the real-time polymerase chain reaction (RT-PCR) assessment of Porphyromonas gingivalis, Prevotella intermedia, Tannerella forsythia, Fusobacterium nucleatum, Treponema denticola, and Campylobacter rectus provides the most accurate information about periodontitis, its progression [47], and its responsiveness to administered treatment [48], while the diagnostic value of Aggregatibacter actinomycetemcomitans currently remains controversial.

Histopathological markers are not routinely used in periodontal diagnosis, but they may provide important information regarding the disease’s nature, pattern of progression, and grade and related to the validation of biomarkers most suitable for everyday use in the clinical setting.

## 5. Shift to Precision Periodontics: Where Are We Stuck, and How Do We Proceed?

New technologies for comprehensive biological profiling of patients have initiated a switch to precision periodontics [49]; however, this remains in the very initial stage. As mentioned above, the driving forces of precision medicine are validated biomarkers and machine learning algorithms, and so far, in periodontology, there is no biomarker validated for diagnostic use, while algorithms are predominantly exploited in observational studies (regression methods and clustering analyses) and rarely for diagnostic purposes. The confusing aspect of the lack of diagnostic markers in periodontology and implantology [34,50,51], despite so many reported biomarker studies, relates to a frequent misinterpretation of biomarkers specifically validated for diagnostic use. Although a biomarker as an indicator of a biological process can be used to study the characteristics of physiological or pathological conditions and their respective responses to different factors (such as treatment), biomarkers validated for diagnostic use need to comply with specific diagnostic requests defined in rigorous guidelines for biomarker validation, varying for each biomarker subgroup [26,52,53]. Biomarker validation studies aim to identify promising candidate markers to answer specific clinical requests, to standardize pre-analytical protocols (sampling and storage) and analytical protocols (laboratorial methods), and to provide highly accurate interpretation systems. Biomarkers validated for diagnostic use need to have a highly reproducible diagnostics protocol with established accuracy, sensitivity, specificity, false positive rate, false negative rate, and diagnostic range precisely disclosed in the diagnostic information; so far, these kinds of diagnostic studies have been scarce in periodontology and implantology [47,51,54]. Indeed, the available studies provide robust information on candidate markers and their capacity to provide specific diagnostic information in periodontal practice. In that context, future diagnostic studies designed according to referent guidelines and to address the gaps observed in the reported studies should easily progress and yield diagnostic markers for periodontal diseases.

Some common reasons for the lack of diagnostic markers in periodontology can be summarized as follows:

- High interstudy variability in clinical diagnostic criteria and case definition;
- Small sample sizes;
- Inappropriate study designs regarding candidate marker selection for specific diagnostic needs, clinical strategy, and data processing;
- Variability in specimen collection and storage protocols (pre-analytical variability);
- Variability in analytical methods (intra-analytical variability);
- Interpretation and data reporting (post-analytical variability).

To address the most frequent limitations encountered in the reported studies, the first step in accelerating the implementation of a precision periodontics approach concerns the rigorous design of biomarker studies according to referent recommendations, starting from the appropriate selection of candidate markers for requested diagnostic information. Regarding microbiological markers, diagnostic focus should be directed to the assessment of a panel of key-stone periopathogens, together with opportunists; this may provide specific diagnostic information on shifts in microflora, recognized as a crucial diagnostic indicator in distinguishing health from disease, disease progression, and responsiveness to the performed treatment. It is thus expected that metagenomic and metatranscriptomic methods, together with culturomics [55], will contribute to the identification of highly specific microbiological markers for accurate diagnosis and prognosis of periodontal disease. In the context of assessing quantitative changes, quantitative RT-PCR is the method of choice, while quantitative omics-based methods are in a stage of development as well.

In the context of biochemical markers, proteomics is an analytical method that can contribute to the identification of highly specific cytokine panels, and different multiplexing methods enable the assessment of a great range of protein profiles [51]. Finally, the bone level represents the epicenter of the entire dental implant concept, and there is no method more accurate for real-time assessment of ongoing bone processes than the direct measurement of bone metabolism. The assessment of bone markers showed the most promising diagnostic capacity among all investigated markers in a prior study [42]. BTMs are fast-response markers that reflect the nature and volume of the ongoing bone processes, practically enabling the clinician to visualize the bone status at the molecular level, far before clinical/radiological manifestations; this is valuable for early diagnosis of the conversion of gingivitis into periodontitis, early assessment of patient compliance with the administered treatment, and diagnosis of possible recurrence during maintenance care. Finally, the majority of commercially available diagnostic assays are intended for biological fluids available in larger volumes, which complicates the analysis of GCF, requiring standardization and adjustment of analytical protocols. Special attention should be paid to detailed reporting on analytical protocols to accelerate the optimization and standardization of measurement protocols suitable for periodontal diagnostics. The advanced “omics” methods that can particularly contribute to the identification of new bone markers are metabolomics, particularly regarding identification of the byproducts of bone destruction for real-time assessment of changes in the bone level over time or in response to treatment. Histopathological studies will contribute to biological definitions of the grading criteria of periodontitis. Finally, the entire process of biomarker validation directly depends on the quality of the clinical aspect of diagnostic studies. Hence, strict adherence to the clinical diagnostic criteria and case definitions defined in the referent classification of periodontal conditions is the ultimate precondition for accurate validation of periodontal biomarkers. Further, studies aiming to validate surrogate endpoints for the assessment of periodontal treatment outcomes should be first conducted according to guidelines for standard periodontal treatment [52]. When considering such complex multifactorial patterns of periodontal disease, combined assessment of clinical parameters with multiple biomarkers expectedly provides more accurate diagnostic information [47], and the best-performing methods for the identification of highly specific biomarker interactions with clinical parameters and their implementation in interpretable fine-tuned clinical diagnoses are machine learning algorithms [24,53]. A common reason for the gap between biomarker research and implementation in clinical practice is also the lack of appropriate tools for interpreting comprehensive panels [25]—another reason why future diagnostic studies in periodontology should consider the integration of algorithms into the study design.

Precision periodontics undoubtedly represents the future of high-quality periodontal care, so it is of paramount importance that future research studies strictly adhere to the recommendations for the validation of biomarkers in order to accelerate the process of their implementation in routine clinical practice. The coordinated collaborative work of an interdisciplinary and international consortium could substantially accelerate the implementation process of precision periodontics, assuring high-quality, reproducible research in larger pooled samples and subsequent fast implementation in clinical practice. Moreover, future studies should focus on the development of biomarker assessment protocols applicable in everyday practice, such as point-of-care testing (POCT), which is still in the developmental stage in periodontology [54,55]. Additionally, the social patterning aspect of periodontal disease should not be neglected, and in this context, the development of patient self-testing methods should be seriously considered as well.
